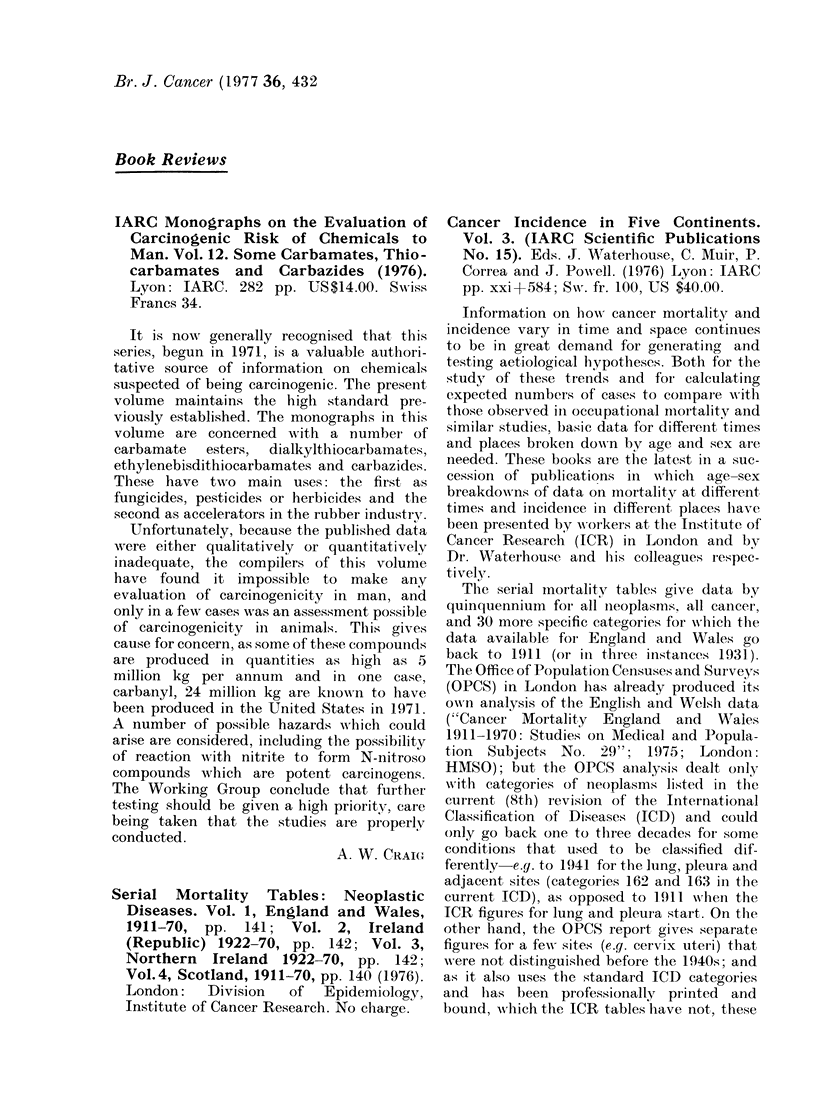# IARC Monographs on the Evaluation of Carcinogenic Risk of Chemicals to Man. Vol. 12. Some Carbamates, Thiocarbamates and Carbazides (1976)

**Published:** 1977-09

**Authors:** A. W. Craig


Br. J. Cancer (1977 36, 432

### *Book Reviews*

**IARC Monographs on the Evaluation of Carcinogenic Risk of Chemicals to Man. Vol. 12. Some Carbamates, Thiocarbamates and Carbazides (1976).** Lyon: IARC. 282 pp. US$14.00. Swiss Francs 34.

It is now generally recognised that this series, begun in 1971, is a valuable authoritative source of information on chemicals suspected of being carcinogenic. The present volume maintains the high standard previously established. The monographs in this volume are concerned with a number of carbamate esters, dialkylthiocarbamates, ethylenebisdithiocarbamates and carbazides. These have two main uses: the first as fungicides, pesticides or herbicides and the second as accelerators in the rubber industry.

Unfortunately, because the published data were either qualitatively or quantitatively inadequate, the compilers of this volume have found it impossible to make any evaluation of carcinogenicity in man, and only in a few cases was an assessment possible of carcinogenicity in animals. This gives cause for concern, as some of these compounds are produced in quantities as high as 5 million kg per annum and in one case, carbanyl, 24 million kg are known to have been produced in the United States in 1971. A number of possible hazards which could arise are considered, including the possibility of reaction with nitrite to form N-nitroso compounds which are potent carcinogens. The Working Group conclude that further testing should be given a high priority, care being taken that the studies are properly conducted.

A. W. CRAIG